# Methylphenidate Promotes a Frontoparietal-Dominant Brain State Improving Cognitive Performance: A Randomized Trial

**DOI:** 10.1523/JNEUROSCI.1693-24.2025

**Published:** 2025-03-18

**Authors:** Weizheng Yan, Şükrü Barış Demiral, Dardo Tomasi, Rui Zhang, Peter Manza, Gene-Jack Wang, Nora D. Volkow

**Affiliations:** National Institute on Alcohol Abuse and Alcoholism, Laboratory of Neuroimaging, National Institutes of Health, Bethesda, Maryland 20892

**Keywords:** brain dynamics, fMRI, methylphenidate, PET, visuospatial

## Abstract

Methylphenidate (MP) is a widely used stimulant medication for the treatment of attention-deficit hyperactivity disorder that enhances brain dopamine signaling and improves attention. However, how dopamine stimulation alters brain state dynamics to support improved attention during task performance is still unclear. To address this, we employed a multimodal neuroimaging approach combining positron emission tomography, functional magnetic resonance imaging, and behavioral tasks, to discover associations between dopamine signaling, brain dynamics, and cognition. Multimodal images were collected from 37 healthy adults under a single-blind, counterbalanced, placebo-controlled crossover study. Dynamic functional analysis was used to compare the alterations in dynamic features of brain states before and after MP. Subsequently, we analyzed the correlation between these brain state changes and baseline striatal D_1_ and D_2_ dopamine receptor (D_1_R, D_2_R) availability. We also examined alterations in dynamic brain states and their effects on visuospatial tasks. The results showed that MP primarily affected frontoparietal-dominant activated (FPN+), somatomotor-dominant activated (SOM+), and visual-dominant suppressed (VIS−) brain states. Specifically, the dwell time and fractional occupancy exhibited significant increases within the FPN+ and VIS− and an opposite trend within the SOM+. Furthermore, the increase of dwell time in FPN+, which was positively correlated with baseline striatal D_1_R availability, was also associated with quicker response in the 2-ball-track task, but not significantly for the 3-ball-track task. The findings suggest that MP's enhancement of brain states with FPN+ and VIS− while decreasing SOM+, in part through D_1_R signaling might underlie MP's improvement of attention for low demanding tasks in healthy populations.

## Significance Statement

Methylphenidate (MP) is primarily prescribed for attention-deficit/hyperactivity disorder, but it is also misused as a cognitive enhancer by individuals seeking to improve cognitive performance. Using advanced brain imaging and behavioral tasks, this study investigates how MP affects dopamine signaling, brain activity, and cognitive performance. Our results demonstrate that MP promoted a frontoparietal-dominant brain state which linked to improved task performance and D_1_ receptor availability. This research also introduces a multilevel neuroimaging approach to studying drug effects, offering a foundation for tailoring interventions by predicting individual variations in responses to medicine.

## Introduction

Methylphenidate (MP) is a stimulant drug that elevates extracellular dopamine (DA) levels in the brain by blocking DA transporters (Butcher et al., 1991; Woods and Meyer, 1991; During et al., 1992; Kuczenski and Segal, 1997; Volkow et al., 2001; Volkow et al., 2012). MP is primarily prescribed for attention-deficit/hyperactivity disorder (ADHD), but it is also misused as a cognitive enhancer by individuals seeking to improve cognitive performance (Teter et al., 2006). However, the actual capabilities and mechanisms of MP as a cognitive-enhancing drug remain a subject of debate (Kortekaas-Rijlaarsdam et al., 2019; Westbrook et al., 2020; Bowman et al., 2023). For example, Bowman et al. observed that the so-called “smart drugs,” such as MP, boost motivation but reduce the quality of effort needed for solving complex problems (Bowman et al., 2023).

PET imaging studies have revealed impaired striatal dopaminergic signaling (reduced D_2_R availability and DA release) in adults with ADHD (Volkow et al., 2007) in association with reduced motivation and inattention (Volkow et al., 2011). In ADHD adults, MP-induced enhancement of DA signaling was associated with improvements in motivation (Volkow et al., 2004) and inattention (Volkow et al., 2012). In healthy adults, MP increased cognitive motivation more for participants with lower baseline DA synthesis capacity (measured using ^18^[F]DOPA PET; Westbrook et al., 2020). Meanwhile studies in healthy adults showed that D_2_R availability in striatum predicted subjective responses to MP, such that larger effects from MP were reported by individuals with higher D_2_R availability (Volkow et al., 1999). To our knowledge, no studies have been reported on the association between D_1_R availability and MP effects. Nonetheless the prior findings collectively suggest that DA enhancing drugs such as MP would be most effective in individuals with DA deficiencies. Thus, we hypothesized that baseline DA receptor availability (D_1_R and D_2_R) would be associated with intersubject variations in MP's effects on cognitive tasks (Volkow et al., 2002b; Froehlich et al., 2011) such that individuals with higher baseline receptor availability would be more sensitive to MP due to an increased capacity for dopamine signaling enhancement.

Our previous study in healthy controls (Manza et al., 2022) revealed that after MP administration, brain activity [measured by the fractional amplitude of low-frequency fluctuations (fALFF)] increased in association cortices and decreased in sensorimotor cortices. Additionally, the within-network resting-state functional connectivity strength decreased more in sensorimotor than in association cortices. These results suggest that MP has different effects on association than on sensorimotor networks. However, the measures of fALFF or of static functional connectivity assume that brain functional connectivity is constant over time, potentially missing additional dynamic patterns of brain function. Previous studies have provided evidence that brain state dynamics are hierarchically organized in time (Breakspear, 2017; Vidaurre et al., 2017; Cornblath et al., 2020) and that incorporating brain dynamic analysis allows for a more comprehensive elucidation of a drug's impact on brain function (Cornblath et al., 2020; Mizuno et al., 2022; Cai et al., 2023). However, the previous studies have not established a clear pathway linking DA levels to brain dynamics and, subsequently, cognitive performance.

Here, we hypothesized that MP alters brain functional activity by increasing striatal DA levels, thereby affecting behavioral performance. This multimodal study aimed to employ dynamic analytical methods to explore how DA receptor (D_1_R and D_2_R) availability and striatal DA levels impact brain dynamics and consequently affect cognitive and attention performance in healthy adults. The study had three specific aims: (1) to analyze the effects of MP on functional dynamics in healthy adults; (2) to examine the relationship between changes in dynamic features and visuospatial task performance; and (3) to investigate the correlation between functional dynamic alterations and DA receptor availability.

## Materials and Methods

**Figure 1.**
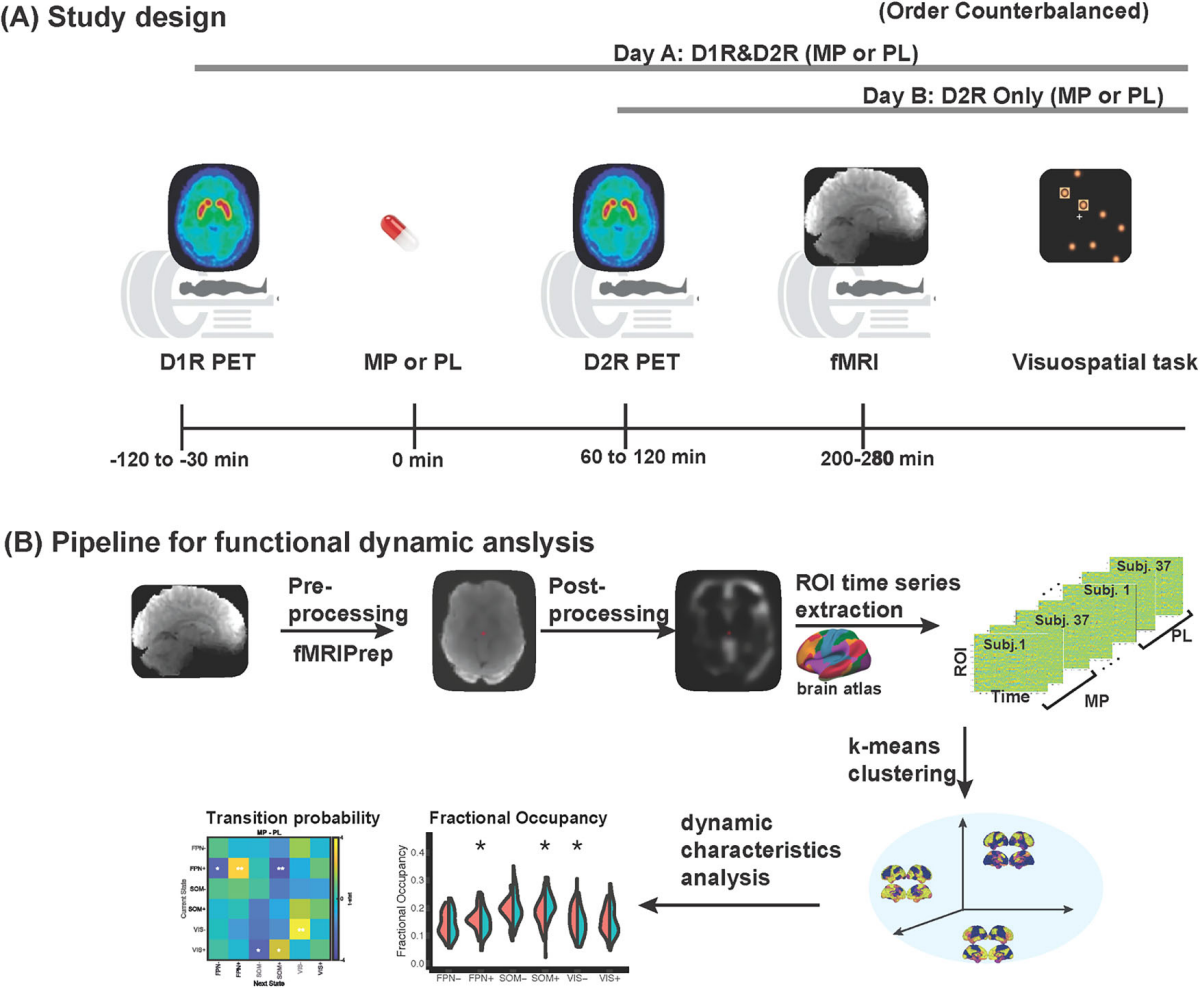
Study design and pipeline for functional dynamic analysis. ***A***, Study design. On the first day, participants underwent a [^11^C]NNC-112 scan to assess D_1_R availability at baseline. Then, participants took the oral medication (either 60 mg MP or placebo) and underwent a [^11^C]raclopride scan to assess D_2_R availability, a resting state fMRI scan to assess brain activity, and a ball-track visual attention task. On the second day, the D_2_R, resting-state fMRI scans, and ball-track tasks were repeated after the other oral drug was given (MP/placebo session order was counterbalanced). ***B***, fMRI dynamic analysis pipeline: multi-echo-multi-band fMRI was preprocessed using fMRIPrep (Esteban et al., 2019) and postprocessed using in-house codes (see Materials and Methods). The ROI time series were extracted from 7 cortical networks (200 ROIs; Schaefer et al., 2018) and 1 subcortical network (32 ROIs; Tian et al., 2020). The extracted ROI time series were concatenated across time and clustered using the *k*-means algorithm for identifying brain state centroids. Then, the dynamic characteristics are then derived and compared between pre- and post-MP.

As shown in Figure 1, we used PET and fMRI, along with behavioral tests, to investigate the relationships between neurotransmitters, brain activity, and cognitive performance. Thirty-seven participants were enrolled in a single-blind, placebo-controlled, crossover study (clinical trial Identifier #NCT03190954, clinicalTrials.gov). Each participant underwent PET and fMRI imaging sessions before and after oral administration of 60 mg MP or placebo in a randomly counterbalanced order. PET imaging for D_2_R availability was acquired twice both for the PL and the MP sessions, whereas PET imaging for D_1_R availability was acquired only once without any drug medication. We analyzed changes in dynamic brain features (e.g., fractional occupancy, dwell time, and appearance rate) associated with MP administration. In addition, we examined the relationship between these dynamic changes, DA receptor availability, and performance on a visuospatial attention task.

### Participants and study design

Data from 37 healthy adults (24 males, 13 females, aged 22–64 years) were included. All participants provided written informed consent, and the study was approved by the Institutional Review Board Committee of the National Institutes of Health. Participants were excluded if they had a history of substance misuse or dependence (other than nicotine), psychiatric disorders, neurological disease, medical conditions that may alter cerebral function (i.e., cardiovascular, endocrinological, oncological, or autoimmune diseases), current use of prescribed or over-the-counter medications, and/or head trauma with loss of consciousness >30 min. Detailed demographic information is shown in Table 1.

**Table 1.** The demographic characteristics of the participants

	Scanner 1: HRRT	Scanner 2: PET/CT	*t*(dt), *p*
Age			
Min-max	33–64	22–61	2.43(35), 0.021
Mean ± SD	48.4(9.6)	37.6(12.5)	
Sex			
*n*, female (%)	7(41%)	6(30%)	0.5037(1), 0.48
BMI			
Min-max	21–39	21–33	0.49(35), 0.63
Mean ± SD	27.2(5.1)	27.9(3.5)	
IQ			
Min-max	79–139	97–129	2.85(35),0.007
Mean ± SD	122.4	109.6	
Race			
*n*, White (%)	11 (65%)	5 (25%)	-
*n*, Black (%)	6 (35%)	10 (50%)	-
*n*, Asian(%)	0 (0%)	3 (15%)	-
n, Other(%)	0 (0%)	2 (10%)	-

Notes: BMI, body mass index; IQ, intelligence quotient; SD, standard deviation.

### MP administration and PET acquisition

PET scans were used to measure the availability of D_1_R using [^11^C]NNC-112 (obtained once at baseline) and D_2_R using [^11^C]raclopride (obtained twice, once after PL and another after MP). Participants were imaged using two scanners based on availability: a Siemens AG High Resolution Research Tomography (HRRT) scanner used for 17 individuals (including 7 females), and a Siemens AG Biograph PET/CT scanner used for 20 individuals (including 6 females). Specifically, all [^11^C]NNC-112 scans were scheduled at 10 A.M. under baseline conditions. The [^11^C]raclopride scans were conducted on two separate occasions for each participant: one performed 1 h after placebo (oral pill) and the other performed 1 h after MP (60 mg oral), in a single-blind manner with counterbalanced session order. The two [^11^C]raclopride scans were performed ∼1 P.M. using the same scanner for each individual to ensure consistency. The technical details for scan procedures were as previously published (Zhang et al., 2021). Briefly, the [^11^C]NNC-112 imaging began immediately after injection of a maximum dose of 555 MBq followed by a series of 21 dynamic emission scans from time of injection to 90 min postinjection. The [^11^C]raclopride imaging was started after an injection of a maximum dose of 370 MBq followed by a series of 22 dynamic emission scans from time of injection to 60 min postinjection. Before analysis, all dynamic emission scan images underwent a rigorous evaluation by one of the investigators (SBD) to ensure exclusion of any images compromised by motion artifacts or misplacement.

### PET modeling, harmonization, and striatum extraction

The MAGIA toolbox (Karjalainen et al., 2020) was employed for PET modeling. MAGIA consisted of frame alignment (motion correction, using the middle image of the image volume time series as master image for the alignment) and coregistration with the individual brain MRI and a quality report for image quality evaluation. To mitigate scanner-specific effects between HRRT and PET/CT, we used ComBat (Fortin et al., 2018), a data harmonization approach, to perform voxel-level harmonization independently for each tracer in the PET assessments to ensure data consistency between the two PET scanners. The ComBat model included age, gender, and race as covariates. The harmonized PET no longer exhibited significant scanner-related differences. For voxel-wise analysis, a subcortical atlas (Tian et al., 2020) consisting of 32 subcortical regions was used to extract voxels in the striatum, including putamen, nucleus accumbens (NAc), and caudate. For ROI analysis, the same subcortical atlas was used to extract and average striatal ROIs.

### MRI acquisition

Approximately 200 to 280 min after MP or placebo administration, participants underwent a resting-state fMRI using a 3.0 T 32-channel Siemens Prisma scanner. For this purpose, a multiecho, multiband EPI sequence was used: multiband factor, 3; anterior-posterior phase encoding; TR, 891 ms; echo times, 16, 33, and 48 ms; flip angle, 57°; 45 slices with 2.9 × 2.9 × 3.0 mm voxels and 520 time points while the participant relaxed with their eyes open (total acquisition time, 8 min). A fixation cross was presented on a black background under dimmed room lighting using a liquid-crystal display screen (BOLDscreen 32, Cambridge Research Systems). The 3D MP-RAGE (TR/TE, 2,400/2.24 ms; FA, 8°) and variable flip angle turbo spin-echo (Siemens SPACE; TR/TE, 3,200/564 ms) pulse sequences were used to acquire high-resolution anatomical brain images with 0.8 mm isotropic voxels field-of-view (FOV), 240 × 256 mm; matrix, 300 × 320; and 208 sagittal slices.

### Functional MRI processing

Resting-state fMRI was preprocessed using fMRIPrep (Esteban et al., 2019) and in-house codes. Specifically, the fMRIPrep pipeline was used for multiecho-multiband optimization, gradient distortion correction, rigid body realignment, field map processing, and spatial normalization to standard MNI space. For postprocessing steps, to enhance the signal-to-noise ratio and maintain consistency with prior studies (Singleton et al., 2022), spatial smoothing was applied with a full-width at half-maximum (FWHM) of 5 mm. The fMRI data were filtered with a bandpass filter ranging from 0.01 to 0.08 Hz. The data also underwent a rigorous detrending process and regression analysis to mitigate the influence of six motion-related and three anatomical-related nuisance variables including white matter, cerebrospinal fluid, and global signal. Time points were excluded if the volume-to-volume BOLD signal met the following criteria: DVARS >150 or framewise displacement (FD) >0.5 mm. Time series for 200 cortical regions (Schaefer et al., 2018) and 32 subcortical regions (Tian et al., 2020) were then extracted and demeaned.

### Dynamic functional analysis

The most fundamental step of dynamic function analysis is to partition the fMRI time series to identify the cluster attribution of each time point. Following the approach of Cornblath et al. (2020) and Singleton et al. (2022), we first extracted the ROI-averaged fMRI time courses from the preprocessed BOLD signals. We then concatenated the time courses of all subjects under both MP and placebo conditions across time. Next, the k-means clustering algorithm was employed to identify clusters of brain activation patterns or states. k-means was implemented using MATLAB (2022b) with embedded function “*kmeans*” with “*correlation*” as the distance metric, calculated as 1 min the sample correlation between points treated as sequences of values. The clustering process was repeated 50 times with random initialization to select the best partitions of the data. The optimal number of clusters was selected based on the elbow criterion. Specifically, we plotted the explained variance curve from *k* = 3 to *k* = 22 and identified the inflection point or “elbow.” Given the increased *k* beyond 6 resulted in <1% variance gain, the *k* in this study was set at 6, considering its balanced and interpretable nature. To validate the robustness of the partitions when *k* = 6, we independently replicated the clustering process 10 times and compared the Adjusted Mutual Information (AMI) across the 10 resulting partitions. The partition with the highest cumulative AMI relative to the others was selected. The dynamic metrics were subsequently calculated. These metrics include transition probability, which refers to the probability of transitioning from one brain state to another over time; fractional occupancy, which describes the proportion of time that the brain spends in a particular state out of the total observation time; dwell time, which refers to the duration that the brain remains in a single state before transitioning to another; and appearance rate, which indicates how frequently a particular state appears throughout the observation period.

### *N*-ball-track task

We used a blocked visual attention paradigm (Rustichini et al., 2009) focusing on sustained attention and visual indexing. Specifically, a limited set of visual objects is marked for rapid attentional processing. Each TRACK epoch, lasting 1 min, consists of five tracking and response intervals. Briefly, a subset of balls (2 or 3 out of 10) is highlighted, followed by their random movement in the visual field. Participants were required to fixate on the central cross and to track these target balls as they moved. At the end of the tracking periods, the balls stopped moving, a new set of balls was highlighted, and participants were instructed to press a button if the highlighted balls were the target balls. Each “DO NOT TRACK” or control epoch consisted of similar 1 min intervals with no ball highlighting. Participants passively watched the balls move and stop. Each task variant (with 2 or 3 balls) contains three cycles of “TRACK” and “DO NOT TRACK” epochs, totaling 6 min and 10 s. The tasks were displayed using MRI-compatible goggles connected to a computer. We recorded response time and hitting accuracy via button presses and synchronized the paradigm with the fMRI acquisition using a scanner trigger signal. The response time was calculated as the average of all button-press timings. For the 2 d scanning sessions, each day followed a fixed sequence with the 2-ball track task first, followed by the 3-ball track task. Of note, although task fMRI was acquired during the behavioral task, this study focused on examining the relationships between DA signaling, brain dynamics (derived from resting state fMRI), and behavior performance. Therefore, only behavior data from this task were used in the analysis.

## Results

### MP promoted frontoparietal network and decreased somatomotor network dominant brain states in healthy adults

**Figure 2.**
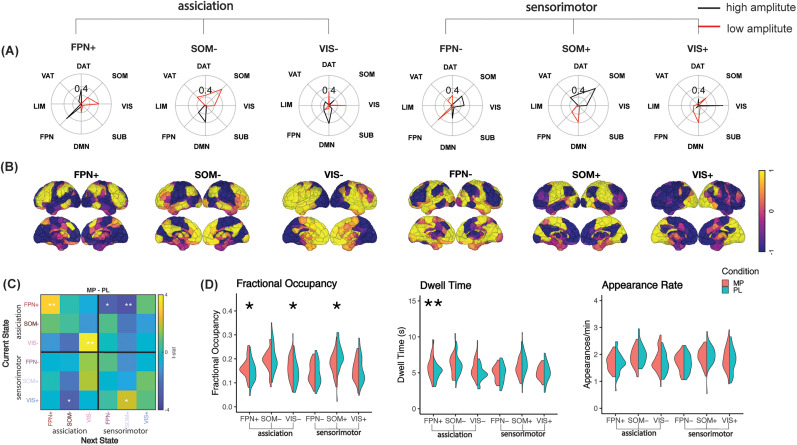
Dynamic analysis results. ***A***, Group average recurrent brain states are represented by the mean activation pattern across all subjects and conditions for each of the six clusters. ***B***, The rendered brain volumes of the six brain states. ***C***, *t* test group comparison of transition probability between MP and PL. ***D***, Dynamic features comparison. Notes: Comparisons in ***C*** and ***D*** were made using two-sided *t* tests and *p* values were corrected for Benjamini–Hochberg multiple comparisons where * represents *p* < 0.05, uncorrected, and ** represents *p* < 0.05, BH corrected. Please note that unless otherwise specified as BH corrected, the reported *p* is before BH correction.

As shown in the radial plots of Figure 2*A*,*B*, after k-means clustering, six cluster centroids, each representing a brain state, were identified. The name of each brain state was assigned by calculating the cosine similarity with eight predefined resting-state networks: frontal parietal network (FPN), limbic network (LIM), ventral attention network (VAT), dorsal attention network (DAT), somatomotor network (SOM), visual network (VIS), default mode network (DMN), and subcortical network (SUB; Yeo et al., 2011; Cornblath et al., 2020; Singleton et al., 2022). Given the regional time courses of each scan were demeaned during fMRI preprocessing, positive centroid values reflect higher than average amplitude, while negative centroid values reflect lower than average amplitude. After k-means clustering with *k* = 6, six brain states are identified including FPN+, FPN−, SOM+, SOM−, VIS+, and VIS−. The dendrogram and Pearson’s correlation coefficients revealed that six brain states could be classified into two groups: one group, comprising FPN+, SOM−, and VIS−, showed high BOLD amplitudes in association cortices and the other comprising FPN−, SOM+, and VIS+ showed high BOLD amplitudes in Sensorimotor cortices. Specifically, the FPN+ brain state consists mainly of high amplitude of FPN and DAT, accompanied by low amplitude of VIS. The SOM+ brain state consists mainly of high amplitude of SOM and low amplitude of DMN. The VIS− brain state consists mainly of low amplitude of VIS and high amplitude of DMN.

After identifying the brain states, we assessed the changes in several dynamic metrics between placebo and MP. As shown in Figure 2*C*, after MP administration, there was a higher tendency for brain states to remain in FPN+ and VIS−, while a lower probability of transiting into the SOM+. Figure 2*D* shows the change in dynamic characteristics after MP administration. Specifically, after MP, fractional occupancy increased in FPN+ (*p* < 0.05) and VIS− (*p* < 0.05), whereas SOM+ decreased (*p* < 0.05). Dwell time increased for FPN+ (*p* < 0.05, BH corrected) and VIS− (*p* < 0.05, BH corrected). There were no significant differences in the appearance rate between placebo and MP administration.

### MP-induced change of FPN+ fractional occupancy and dwell time were associated with faster response in the 2-ball-track task

**Figure 3.**
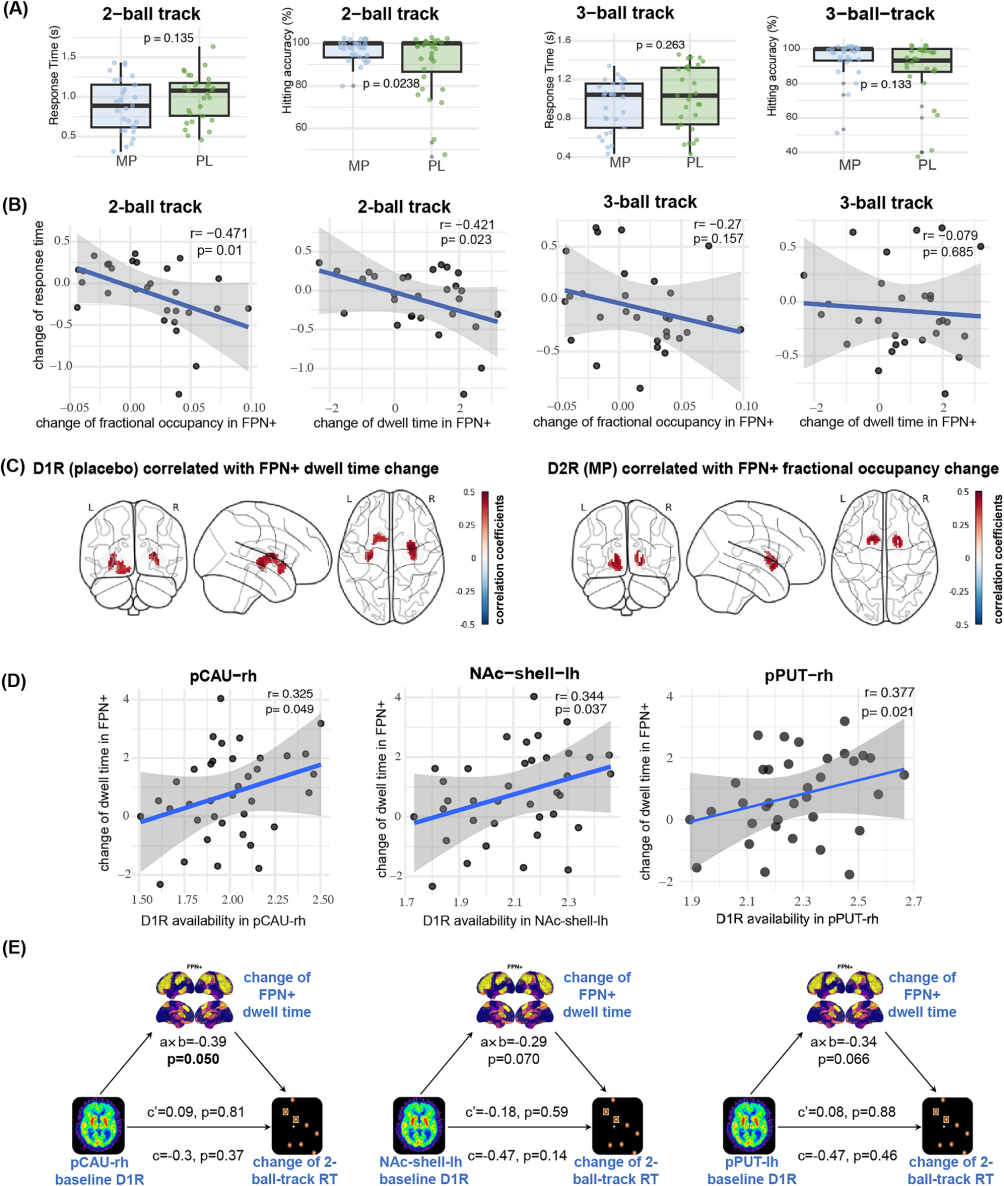
Analysis of the relationship between brain dynamics, performance on ball-track tasks, and dopamine receptor availability. ***A***, Comparison of ball-track performances between MP and Placebo groups. The response time and hitting accuracy were metrics for comparison. Values correspond to means and standard deviations. ***B***, Correlation between dynamic changes induced by MP with the ball-track task response time. ***C***, Voxel-based correlation analysis displays the voxels in the striatal that had significant correlations [*p* (uncorrected) <0.05 with a cluster size of at least 30 spatially contiguous voxels] with the dynamic characteristic change of FPN+ induced by MP. The color bar represents the value of the correlation coefficients. ***D***, ROI-based correlation analysis showed that D_1_R availability in the striatum was positively correlated with the dwell time change of FPN+ after MP. The shaded area represents three times the variance. ***E***, Mediation analysis. *a* × *b*, the indirect effect; *c*, the total effect; *c’*, the direct effect. Notes: pCAU-rh, right hemisphere posterior caudate; NAc-shell-lh, left hemisphere nucleus accumbens shell; pPUT-lh, left hemisphere posterior putamen.

Among the 37 subjects, 29 participants completed the 2-ball-track task, and 31 participants completed the 3-ball-track task under both placebo and MP conditions. We used response time and hitting accuracy as metrics to assess the speed and accuracy of task completion. As shown in Figure 3*A*, generally, after MP administration, there was a trend toward improvement in accuracy and a reduction in response time. The improvements in accuracy reached significance for the 2-ball-track task (mean ± SD: PL: 90.3 *± *14.4%, MP: 97.0 *± *4.9% *p* = 0.023) and though in the same direction for the more demanding 3-ball-track task this was not significant (mean ± SD: PL:89.0 *± *16.4%, MP: 94.8 *± *10.1%, *p* = 0.133).

We further analyzed how changes in brain dynamics caused by MP affected performance in the *n*-ball-track task. Given that significant changes in brain dynamics were observed after MP administration, we conducted a correlation analysis between changes in task performance and the dynamic characteristics significantly altered by MP (including fractional occupancy of FPN+, SOM+, VIS−, and dwell time of FPN+ and VIS−). The results indicated a significant correlation only between changes in FPN+ and changes in response time. Specifically, as shown in Figure 3*B*, in the 2-ball-track task, increases of dwell time (*r* = −0.42, *p* = 0.02) and fractional occupancy (*r* = −0.47, *p* = 0.05) in FPN+ significantly correlated with the changes in response time. However, in the 3-ball-track task, the correlation between FPN+ change and response time change was not significant. Regression analysis also indicated that intelligence quotient (IQ) effects were negligible for all these correlations.

### Baseline D_1_R availability in striatum was correlated with MP-induced dwell time change in FPN+ and indirectly affected the 2-ball-track task response time

We hypothesized that MP alters brain functional activity by increasing striatal DA levels, thereby affecting behavioral performance. Consequently, we predicted that individual differences in DA receptor availability would modulate the responses to MP. To test this, we first conducted a voxel-level correlation analysis between striatal DA receptor availability features and fMRI-derived dynamic features. This voxel-wise approach was chosen to identify the overall correlated voxels and was subsequently complemented with the ROI-based analysis. The DA receptor availability features included baseline striatal D_1_R availability, striatal D_2_R availability after placebo, and striatal D_2_R availability after MP. The fMRI-derived dynamic features were FPN+ dwell time, FPN+ fractional occupancy, VIS− dwell time, VIS− fractional occupancy, and SOM+ fractional occupancy. These five dynamic features were selected because they exhibited significant MP-PL group differences.

As shown in Figure 3*C*, the MP-induced dwell time change in FPN+ exhibited strong positive correlation with baseline striatal D_1_R availability. The MP-induced fractional occupancy changes in VIS− exhibited positive correlation with D_2_R availability after placebo whereas the fractional occupancy changes in FPN+ exhibited a positive correlation with D_2_R after MP.

For the ROI level correlation analysis, we used a subcortical atlas-based partition (Tian et al., 2020). The correlations between the ROI-level measures of D_1_R and D_2_R availability and the dynamic features are shown in Figure 3*D*. These results, which are consistent with the voxel-based findings, showed a significant correlation between FPN+ dwell time change and D_1_R availability in the right posterior putamen (*r* = 0.38, *p* = 0.027), left NAc-shell (*r* = 0.34, *p* = 0.037), and right posterior caudate (*r* = 0.33, *p* = 0.05). Although not significant, the correlations in the opposite hemisphere showed the same trend with left posterior putamen (*r* = 0.31, *p* = 0.06), right NAc-shell (*r* = 0.26, *p* = 0.11), and left posterior caudate (*r* = 0.23, *p* = 0.16). Regression analysis also indicated that IQ effects were negligible for all these correlations.

Although the correlation between striatal D_1_R availability and performance in the 2-ball-track task was not significant, striatal D_1_R availability showed a strong correlation with FPN+ dynamics, which, in turn, exhibited a significant correlation with the 2-ball-track task. Mediation analysis was performed to determine whether FPN+ dynamics acted as a mediator for the two-ball-track task. Mediation analysis was performed using the “mediation” package in *R* (version 4.5.1). Specifically, striatal D_1_R availability was designated as the independent variable, and the MP-induced change in response time for the two-ball-track task was the dependent variable. The MP-induced change in FPN+ dwell time served as the mediator. The significance of mediation effects was assessed using 1,000 bootstrap iterations. Mediation analysis revealed a significant indirect effect (*a* × *b*) of baseline striatal D_1_R on the MP-induced change in 2-ball-track response time. Specifically, pCAU-rh showed significance (*p* = 0.05), while NAc-shell-lh and pPUT-lh exhibited trends (*p* = 0.07).

## Discussion

Establishing the connections between DA signaling, brain functional dynamics, and cognitive performance enabled us to uncover the effects of MP on the brain at multiple levels. In this study, we utilized a comprehensive set of measurements, including PET, fMRI, and behavioral tests. Dynamic functional analysis revealed a significant enhancement in FPN+ and VIS− following administration of MP (60 mg orally). The MP-induced changes in these dynamic features showed significant correlations with striatal DA receptor availability. Specifically, the change in FPN+ dwell time was positively correlated with response time in the 2-ball-track task, though no significant correlation was observed for the 3-ball-track task, which is consistent with the limited cognitive benefits of MP in healthy individuals (Repantis et al., 2021). In summary, dynamic functional methodologies allowed us to identify a relationship between striatal D_1_R availability and task performance after MP, mediated mainly by FPN+ dynamics. We composed a song (Audio 1) using SUNO, an AI music generator, encapsulating the main findings of this study. The song is also available at https://suno.com/song/3623d63f-7de7-42fc-8fd3-cd5fa5c419f4.

**audio 1.**
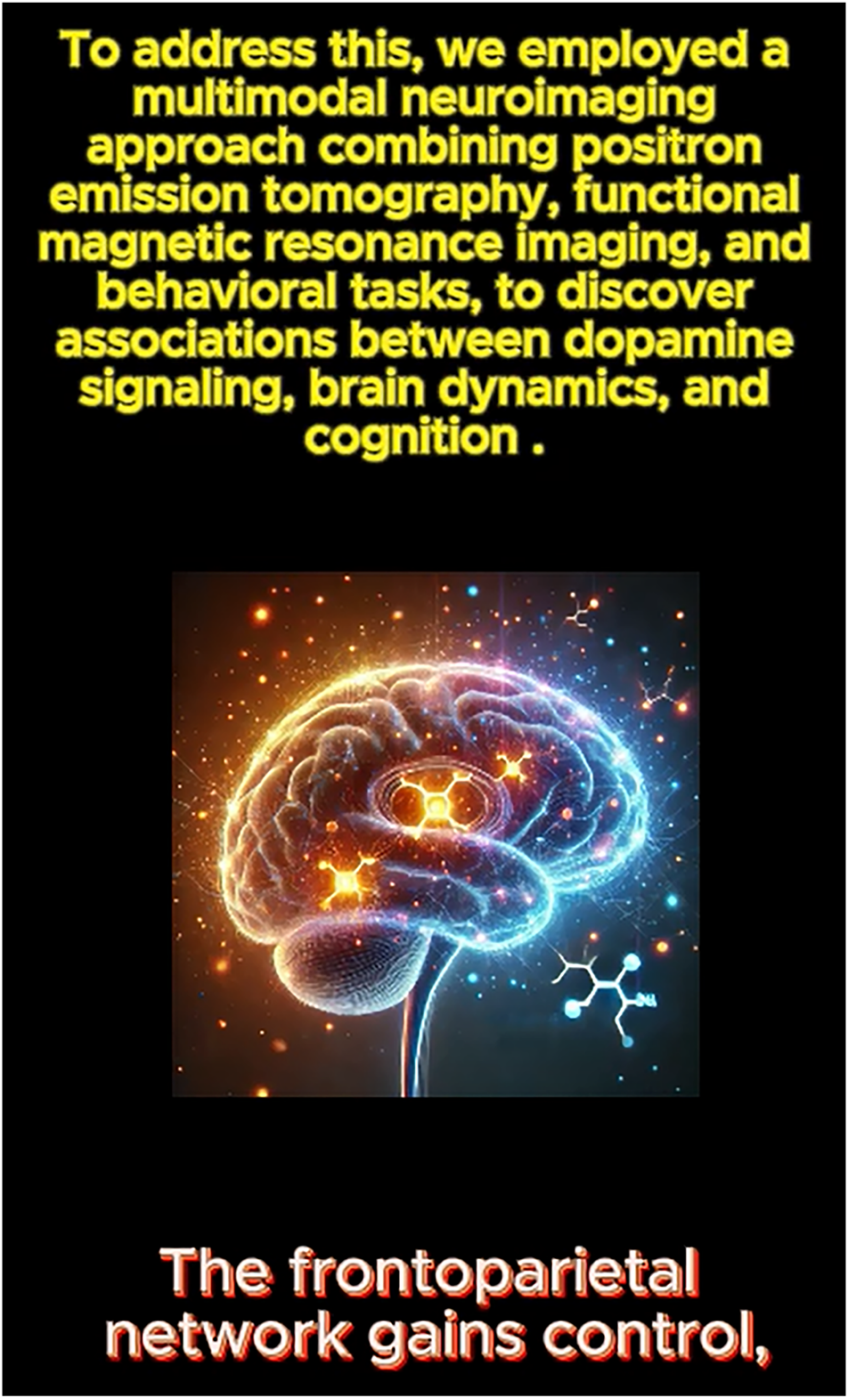
A song composed using SUNO, an AI music generator, encapsulating the main findings of this study. The song is also available at https://suno.com/song/3623d63f-7de7-42fc-8fd3-cd5fa5c419f4

10.1523/JNEUROSCI.1693-24.2025.d1 Audio 1 A song composed using SUNO, an AI music generator, encapsulating the main findings of this study. The song is also available at https://suno.com/song/3623d63f-7de7-42fc-8fd3-cd5fa5c419f4. Download Audio 1, MP4 file.

Different from traditional functional connectivity methods that primarily emphasize the synchronicity between brain regions, dynamic functional connectivity reveals changes in dynamics characteristics such as transition probability, fractional occupancy, dwell time, and appearance rate of various functional network activities. From a dynamical perspective, the most prominent finding was an increase of fractional occupancy and dwell time in FPN+ and VIS−, along with a decrease of fractional occupancy in SOM+. In FPN+ and VIS−, the high-amplitude networks were primarily within association networks, whereas in SOM+, the high-amplitude networks were predominantly within sensorimotor networks. These results align with previous static functional connectivity findings that showed increased activity in association networks and decreased activity in sensorimotor networks after MP (Manza et al., 2022).

In each brain state, the DMN consistently appeared in an opposing direction to the SOM and VIS networks. Specifically, when the amplitude of the DMN network was high, the SOM or VIS amplitude were low, and vice versa. These brain states might be influenced by our use of resting-state data for analysis and different brain states might emerge while engaged in various tasks. The FPN+ brain state predominantly consists of high-amplitude FPN and DAT networks. Since FPN and DAT are associated with cognitive processing, an increase of dwell time and fractional occupancy in FPN+ suggests a brain state more likely to be conducive to cognitive efforts and one that is less distracted by visual stimuli or urges to move. Dixon et al. (2018) investigated the FPN's functional connectivity under varying cognitive demands and identified two distinct FPN subsystems: One with stronger connectivity to the DMN, indicating greater involvement in introspective processes, and another with stronger connections to the DAT, suggesting a key role in visuospatial perceptual attention. In our study, following MP administration, participants tended to show quicker responses in the 2-ball-track task that was not significant as there was large intersubject variability in MP effects. However, for individuals in whom MP reduced their reaction time to the 2-ball-track task demonstrated a clear alignment with MP increase of FPN+ dwell time. As such we interpret this to reflect MP's selective impact on cognitive control processes. Interestingly, we did not observe a significant correlation with the more complex 3-ball-track task, suggesting that while MP-induced brain changes can boost cognitive effort (Zametkin, 2010), its effectiveness in more demanding cognitive tasks might be limited (Batistela et al., 2016). However, since the placebo accuracy with the 3-ball-track task was almost 100%, this could have limited our ability to detect any improvement; whereas the placebo accuracy for the 2-ball-track evinced lower performance for some participants, which might have reflected their disinterest in this very simple task and allowed us to observe improvements. This is consistent with Bowman et al.'s findings, where psychostimulants increased motivation but not the quality of effort in complex problem-solving (Bowman et al., 2023), and with studies showing that stimulants are particularly effective in improving performance by enhancing motivation to perform the task (Ilieva and Farah, 2013).

MP's therapeutic effects are in part due to amplification of DA signals and the intersubject variability reported in the response to MP has been shown to be due in part to differences in DA tone and receptor availability between individuals (Volkow et al., 2002a). Thus, measuring baseline DA receptor availability could theoretically predict changes in brain activity caused by MP, a hypothesis that our experiment confirmed. Our correlation analysis showed that the baseline level of striatal D_1_R availability positively correlated with the increase in MP-induced FPN+ dwell time. In addition, although with limited subjects, this exploratory mediation analysis indicates that FPN+ dynamics mediated the relationship between striatal D_1_R availability and visuospatial task performance.

Interestingly, the increase in FPN+ was also associated with striatal D_2_R availability (left NAc-shell, *r* = 0.32, *p* = 0.05) but only for the measure after MP, not after placebo. Since MP reduces striatal D_2_R availability by increasing binding competition with [^11^C]raclopride, as synaptic DA is increased (Volkow et al., 1994), the correlation between D_2_R availability after MP and MP-induced increase in FPN+ fractional occupancy but not for placebo suggests that it reflects changes in D_2_R-mediated signaling.

The association of MP effects on brain dynamics with DA receptor availability could explain why MP is beneficial for ADHD, which is characterized by deficits in dopaminergic singling as evidenced both by decreased D_2_R striatal availability and DA release (Volkow et al., 2007). Notably in individuals with ADHD, the reduction in symptoms of inattention with MP was associated with the increases in striatal DA signaling it triggered (Volkow et al., 2012). Our results also align with previous findings that participants with higher striatal DA synthesis capacity showed greater willingness to exert effort, while MP increased cognitive motivation more in participants with lower synthesis capacity (Westbrook et al., 2020). Our experiment established a significant relationship between D_1_R receptor availability after placebo and D_2_R receptor availability after MP and the predominance of an FPN+ brain state, providing valuable guidance for selecting individuals who would be more likely to benefit from MP to advance personalized treatment approaches.

The study has limitations and potential directions for future research. First, this multimodal study was conducted on healthy adults. Considering that MP is a frontline medication for ADHD, it is crucial to collect and compare multimodal data in ADHD participants. Second, due to equipment limitations, our data collection for PET and fMRI post-MP or placebo administration was conducted separately. As a result, fMRI data were collected ∼200–280 min after drug administration, beyond the peak drug effect (120 min) but still within the period when plasma drug levels were within the therapeutic range after a 60 mg MP dose (Swanson and Volkow, 2002; Spencer et al., 2006). Further studies using simultaneous PET/MR imaging are needed to clarify the associations between MP's pharmacokinetics and its effects on brain functional dynamics. Third, this study focused on D_1_R and D_2_R in the striatum but did not assess cortical DA receptors, which are also involved in MP's effects. This will require studies with PET radioligands with stronger signals in cortical regions. Besides, our measures of D_2_R after MP with [11C]raclopride are confounded by the fact that they reflect both D_2_R levels but also level of endogenous dopamine competing for binding to these receptors. Moreover, MP exerts its pharmacologic effects by increasing both dopamine and norepinephrine signaling in the brain (Faraone, 2018). Future studies are needed to investigate norepinephrine's contribution to MP's effect on brain dynamics. Finally, though we aimed to evaluate the MP's effects on task demands, participants had perfect accuracy for 3-ball-track task while under placebo, indicating that this task was not sufficiently challenging to reveal MP-induced benefits. Besides, our correlation analysis was limited by the small sample size and uncorrected multiple comparison, highlighting the need for future studies with larger sample size and more challenging tasks to validate these results.

In summary, this study investigated the relationship between dopamine receptor availability, the effects of MP on performance in a visuospatial task, and brain state dynamics. The findings established a correlation between MP-induced changes in FPN+ (a brain state with predominant high amplitude of FPN and DAT) and quicker response on an attention task. Furthermore, correlations between baseline D_1_R availability, D_2_R after MP, and MP-induced changes in brain dynamics that favor FPN+ were identified. This study also presents a multilevel neuroimaging approach to investigate drug effects, providing a framework for personalizing medication interventions by predicting individual variations in drug responses such as MP.

## Code Availability

All codes related to the dynamic analysis and regression analysis are shared on GitHub https://github.com/WizardYan/MP_PET_fMRI.
